# Complete Genome Sequences of Two Dengue Virus Serotype 1 Genotype V Strains from Different Lineages

**DOI:** 10.1128/genomeA.01064-16

**Published:** 2016-09-29

**Authors:** Danila Vedovello, Tauyne Menegaldo, Joice M. Biselli-Périco, Leila Sabrina Ullmann, João Pessoa Araújo Junior, Maurício Lacerda Nogueira

**Affiliations:** a Faculdade de Medicina de São José do Rio Preto (FAMERP), São José do Rio Preto, São Paulo, Brazil; b Universidade Estadual Paulista (UNESP), Botucatu, São Paulo, Brazil

## Abstract

Previous phylogenetic studies involving dengue virus serotype 1 (DENV1) have shown several lineages of genotype V circulating worldwide. After sequencing the complete genome of strains from São José do Rio Preto, São Paulo, Brazil, we identified a list of 50 different amino acids that differ between the two lineages, announced here.

## GENOME ANNOUNCEMENT

Dengue virus (DENV) belongs to the *Flaviviridae* family, in the *Flavivirus* genus, and is an arbovirus that commonly circulates worldwide (1). Brazil has experienced epidemics of four dengue serotypes, each circulating in different periods, regions, and at different intensities (2). Specifically for DENV serotype 1 (DENV1), phylogenetic studies of the envelope gene sequences revealed up to 6 different lineages subdividing genotype V (3–6). In the city of São José do Rio Preto (SJRP), São Paulo, Brazil, the circulation of lineages L1 and L6 has been detected since 2012 (7).

We studied some positive isolates for DENV1 detected in this city between 2008 and 2014. DENV1 was detected by multiplex reverse transcription-PCR (RT-PCR), using as a strategy cDNA production by *Flavivirus*-gender-specific primers (8). After positive DENV1 detections, the DNA was transcribed again, now using random primers for DNA library construction and Illumina genome sequencing (9). For this project, 11 complete genomes were sequenced, with two of them described in this announcement, those of strains BR/SJRP/287/2011 and BR/SJRP/287/2011.

The total lengths of the two genomes were 10,699 nucleotides (nt) and 10,917 nt, with 5′–3′ untranslated region (UTR) sequences of 185 to 335 nt and 202 to 563 nt, respectively. In the full DENV1 polyprotein, composed of 3,392 amino acids (aa), 50 characteristic amino acids of each SJRP/DENV1 lineage were observed: C protein, position 95 (Ile/Met); Pr peptide, positions 143 (Val/Ala) and 203 (Asp/Glu); small envelope protein, position 236 (Lys/Arg); M protein, position 252 (Leu/Phe); envelope protein, positions 618 (Leu/Ser), 674 (Lys/Arg), 708 (Leu/Val), and 716 (Ile/Val); nonstructural 1 (NS1) protein, positions 921 (Asp/Gly), 937 (Ile/Gly), 950 (His/Tyr), 999 (Ile/Thr), 1017 (Val/Ile), 1022 (Tyr/Phe), and 1068 (Tyr/Asn); nonstructural NS2 protein, positions 1145 (Ile/Met), 1233 (Val/Ile), 1266 (Asp/Glu), 1282 (Ile/Val), 1283 (Arg/Lys), 1285 (Thr/Ser), 1295 (Met/Val), and 1298 (Ala/Val); serinoprotease NS3 protein, positions 1646 (Thr/Ser), 1656 (Asp/Asn), 1912 (Glu/Asp), and 1949 (Ile/Val); nonstructural NS4A protein, positions 2162 (Val/Ala) and 2183 (Met/Thr); nonstructural NS4b protein, positions 2261 (His/Tyr), 2264 (Val/Ala), 2268 (His/Gln), 2278 (Arg/His), and 2397 (Thr/Ala); and RNA-dependent RNA polymerase NS5 protein, positions 2523 (Lys/Arg), 2628 (Thr/Met), 2863 (Ala/Thr), 3078 (Ser/Asn), 3122 (Ser/Leu), 3133 (Lys/Glu), 3134 (Lys/Arg), 3135 (Val/Ala), and 3282 (Thr/Ala).

The genetic signatures described herein are being explored in other investigations about biological characteristics of DENV1 lineages.

### Accession number(s).

These genomes have been deposited in GenBank under accession numbers KP188540 (BR/SJRP/287/2011 strain) and KP188543 (BR/SJRP/484/2012 strain). The versions described here are the first versions.
